# Prevention of Postoperative Events following Reversal with Sugammadex or Neostigmine (the P-PERSoN Trial): Pilot Data Following Early Termination of a Prospective, Blinded, Randomised Trial

**DOI:** 10.1155/2022/4659795

**Published:** 2022-07-08

**Authors:** Benjamin Olesnicky, Matthew Doane, Clare Farrell, Greg Knoblanche, Anthony Delaney

**Affiliations:** ^1^Department of Anaesthesia, Royal North Shore Hospital, St Leonards, Australia; ^2^The University of Sydney, Sydney, Australia; ^3^Northern Sydney Anaesthetic Research Institute, St Leonards, Australia; ^4^Department of Anaesthesia, Westmead Hospital, Sydney, Australia; ^5^Department of Intensive Care Medicine, Royal North Shore Hospital, St Leonards, Australia; ^6^Division of Critical Care, The George Institute for Global Health, Sydney, Australia

## Abstract

**Background:**

Residual paralysis following anaesthesia is common and can lead to postoperative morbidity. While sugammadex has been shown to be effective in minimising residual paralysis, uncertainty exists as to whether its use reduces any associated morbidity. We designed this trial to determine if the use of sugammadex for the reversal of intraoperative aminosteroid neuromuscular blockade results in improvements in postoperative pulmonary complications, complications in the recovery unit, postoperative nausea and vomiting, and patient satisfaction, when compared to reversal with neostigmine.

**Methods:**

A prospective, double-blind, randomised controlled trial in adult patients admitted for surgical operations at two Australian hospitals between December 2018 and March 2019 was performed comparing the reversal of neuromuscular paralysis using sugammadex 2 mg/kg versus neostigmine 50mcg/kg. Statistical analysis of continuous data was performed using two tailed *t*-tests, with categorical and ordinal data being assessed by chi-squared analysis.

**Results:**

The trial was terminated due to a combination of resource constraints and the 2019 novel coronavirus disease (COVID-19) pandemic. Of 51 patients screened, 33 were eligible for participation and 30 subsequently recruited and randomised. All patients received the intended treatment allocated. Data for the primary outcome was obtained in all patients. There was no difference in the rates of postoperative pulmonary complications between the sugammadex and neostigmine groups (0% (0/19) vs 9% (1/11) RR 5.0 (95% CI 0.22–113) *p*=0.37. There was no difference in any of the secondary outcomes between the groups.

**Conclusions:**

The P-PERSoN trial showed no difference in postoperative pulmonary complications between sugammadex and neostigmine based reversal of aminosteroid neuromuscular block, but was underpowered to show any difference due to early trial termination. The randomisation and data collection was feasible. We support the need for an adequately resourced and funded randomised controlled trial to address this important clinical question.

## 1. Background

The practice of modern anaesthesia involves the use of muscle relaxants to provide muscle paralysis to enable surgical access and to allow effective mechanical ventilation. At the end of surgery, these muscle relaxants need to either be cleared and no longer be active, or be reversed, as significant residual relaxant activity will lead to residual paralysis. Residual paralysis is associated with increased patient morbidity. [1–5].

Sugammadex was introduced into clinical practice in Australia in 2009 and was specifically designed to reverse rocuronium by encapsulating it irreversibly and blocking its activity at the neuromuscular end plate [6]. Previous research has explored the effectiveness of sugammadex to prevent residual paralysis, consistently showing a reduction in the incidence of residual paralysis with sugammadex when compared with neostigmine [5, 7–9].

While sugammadex is shown to consistently reduce the incidence of residual paralysis, studies of the ability of sugammadex to prevent postoperative morbidity provide conflicting evidence. A single published randomised prospective study showed that, while sugammadex was associated with a significant reduction in residual paralysis, this did not lead to a reduction in postoperative pulmonary complications (PPCs) [10].

In addition to this, there have been three recent large trials utilising large data sets, which produced conflicting results. These studies showed that the use of sugammadex either was [11] or was not associated with a reduction in PPCs [12, 13].

These conflicting results provide equipoise as to the clinical effect of sugammadex on postoperative pulmonary complications in modern anaesthetic practice and justify the need for a randomised controlled trial to determine whether the use of sugammadex improves patient outcomes.

P-PERSoN was designed as a prospective multicentre, double-blinded, randomised controlled trial to evaluate if sugammadex use reduced the incidence of postoperative pulmonary complications compared to neostigmine reversal.

## 2. Methods

### 2.1. Trial Design

Across two centres (both a large quaternary public hospital and private hospital setting), adults admitted for surgical operations requiring muscle paralysis were enrolled in a prospective, double-blind, randomised controlled trial comparing specific outcomes following reversal with sugammadex or neostigmine. The study was approved by the Northern Sydney Local Health District Human Research Ethics Committee (RESP/16/289) and the North Shore Private Hospital Ethics Committee [2017-001].

The trial was registered at the Australian New Zealand Clinical Trials Registry (www.anzctr.org.au) # ACTRN12616000063415 (21/01/2016) and the U.S. National Library of Medicine (www.ClinicalTrials.gov) # NCT02825576 (07/07/2016). The trial was compliant with the Australian National Health and Medical Research Council (NHMRC) statement on ethical conduct in human research (2007) [14] and the note for guidance on good clinical practice (CPMP/ICH-135/95) [15]. A preprint of this study was submitted prior to formal publication [16].

### 2.2. Participants

Screening for eligible patients was conducted by examining operating theatre and preadmission clinic schedules for each day. Inclusion criteria were: patients over 18 years of age, planned to undergo noncardiac surgery with an expected operative time of >2 hours, use of endotracheal intubation, and an expected hospital length of stay greater than one night. These criteria were designed to preferentially select patients who would likely be at intermediate or high risk for PPCs [17].

Exclusion criteria were: patients previously recruited, patient weight > 200 kg, patients planned to remain intubated postoperatively, and those with known hypersensitivity reactions or contraindications to any of the study drugs.

Patient recruitment occurred during a preoperative consultation, either in a preadmission clinic or on a hospital ward, prior to their transfer to the operating theatre.

### 2.3. Interventions

Patients were randomly assigned to one of two groups; the sugammadex group or the neostigmine group. At the end of surgery, the participant received a blinded reversal dose via a 10 ml syringe with administration of 1 mL/10 kg of the blinded solution. The solution was standardised to contain the protocol's reversal dose that would equate to either 2 mg/kg of sugammadex or 50 mcg/kg neostigmine with 10 mcg/kg glycopyrrolate.

The study protocol required the use of intraoperative neuromuscular twitch (NMT) monitoring to ensure the return of a train-of-four count (TOFC) greater than or equal to 2 prior to reversal and restricted the use of neuromuscular blockers to rocuronium or vecuronium, at the choice of the individual anaesthetist.

There were no restrictions on the mode of anaesthesia, analgesia (including opioid use), use of postoperative nausea and vomiting (PONV) prophylaxis, or timing of neuromuscular blockade reversal.

### 2.4. Randomisation

Patients were allocated to treatment group via a computer-based simple randomisation software (https://randomizer.org). Group allocations were placed in a sealed opaque envelope by a research team member who was not involved in patient treatment or follow up. Prior to reversal, the treating anaesthetist recruited a member of the hospital clinical staff to open the next available randomisation envelope, draw up the allocated reversal agent based on the enclosed instructions, and deliver this to the treating anaesthetist. This individual was then not involved in further management of the trial patient.

### 2.5. Blinding

The patient, treating anaesthetist, all care providers, and research personnel assessing patient outcomes were all blinded to the patient's treatment allocation.

### 2.6. Outcomes

The primary outcome was the incidence of PPCs (as defined by the European Perioperative Clinical Outcome (EPCO) statement [18] in the first three days postoperatively (Table 1)).

Secondary outcomes were: patient reported quality of recovery at day 1 and 30 utilising the QoR-15 [19], the incidence of PONV, and the incidence of a defined set of airway events in the postanaesthetic care unit (PACU). In addition to the quality-of-recovery-15 score (QoR-15) at day 30, patients were also asked if they had also received either antibiotics or bronchodilators to treat a chest infection (or if previously on bronchodilators, whether they had increased their usage or dose) in the interim. The hospital discharge summary was reviewed to identify the diagnosis of a respiratory infection or associated complications and to calculate hospital length of stay.

Table 1 outlines the EPCO definition, ARISCAT, and Apfel scores, PACU Airway Events and the PONV scoring systems used.

### 2.7. Sample Size

We planned to study 972 based on a power analysis, but as this study was terminated early, the data for 30 patients is presented as pilot data.

### 2.8. Statistical Analysis

The presence of a PPC, and the presence of PACU events were assessed as categorical variables, the QoR-15 score and hospital stay were assessed as continuous variables, and the PONV score was assessed as an ordinal variable. Continuous variables were assessed with two-tailed *t*-tests, categorical and ordinal data were assessed by the chi-squared analysis of contingency tables (or Fisher's exact test when *n* < 5 in any outcome).

## 3. Results

### 3.1. Participant Flow

The trial commenced in December 2018 and was subsequently paused in March 2019 due to the abrupt resignation of research personnel needed to support the trial. This was followed by a prolonged approval and recruitment process to replace them. The recruitment process was completed in January 2020, and while planning to restart, the COVID-19 epidemic occurred. Due to the delay in restarting and absence of funding, the trial investigators made the decision to terminate the trial early and the results were analysed to inform future large-scale randomised controlled trials (RCTs) comparing similar outcomes with the use of sugammadex or neostigmine.

**Figure 1:**
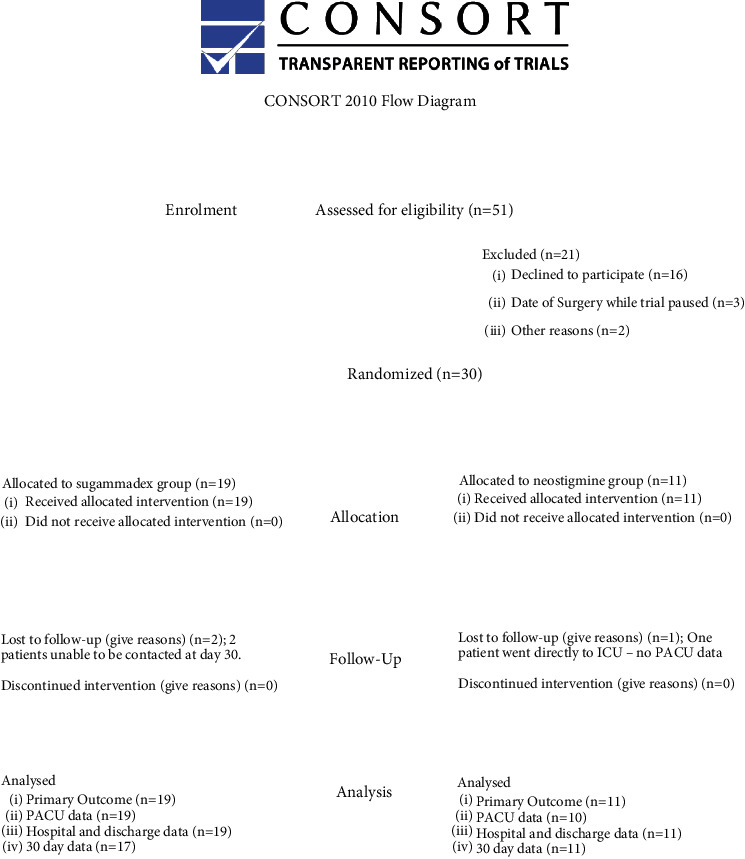
CONSORT flowchart for the P-PERSoN trial.

A total of 51 patients were screened for eligibility. Of those 33 (65% of screened patients) met criteria and were enrolled. There were three patients who had their surgery following study termination, leaving 30 patients to achieve randomisation. The study drug was successfully delivered to 100% of randomised patients. All patients had primary outcome data collected, with a 97% data completion rate for in-hospital data and a 93% completion rate for 30-day data collection (Figure 1). The CONSORT  checklist for this trial is available in the Supplementary Material (available (here)).

PACU stands for postanaesthesia care unit and ICU is the intensive care unit. . Baseline demographics and clinical characteristics were similar in both groups (Table 2).

All randomised patients received the study drug as per their assigned group. There was one violation in the sugammadex group, where a patient received the assigned reversal agent despite having only one twitch on NMT monitoring (reported as being suitable for extubation on clinical grounds by the treating anaesthetist). One patient had no PACU data, as they unexpectedly remained intubated and subsequently were transferred directly to the intensive care unit (ICU) (neostigmine group). Two patients in the sugammadex group were lost to follow up for 30-day data collection.

Due to the early termination and low numbers, there were no interim analyses performed and subgroup analysis was not performed. While the number of participants recruited for participation does not meet those needed to power the study's analysis, we present their results below.

### 3.2. Primary Outcome–Postoperative Pulmonary Complications

One patient in the neostigmine group, and no patients in the sugammadex group, had a documented PPC (9% (95% CI 0–41%): vs 0% (0–17%)). RR 5.0 (95% CI 0.22–113) *p*=0.37.

### 3.3. Quality of Recovery

The neostigmine group reported a mean QoR-15 score (95% CI) of 87 (60–104) at day 1, and 133 (120–146) at day 30, the mean (95% CI) QoR score in the sugammadex group was 102 (88–116) *p*=0.21 and 129 (117–142) *p*=0.61, respectively.

### 3.4. Postoperative Nausea and Vomiting

Postoperative nausea and vomiting was reported in one subject (1/10) in the neostigmine group vs three patients in the sugammadex group (3/19) RR 1.6 (95% CI 0.18–13.3) *p*=0.99.

### 3.5. Complications in the Postoperative Anaesthesia Care Unit

There was one incidence of desaturation to <90% in both the sugammadex and neostigmine groups (5.3% vs 10% *p*=0.99). There were no other airway complications detected in either group. Two patients in each of the groups required a review by an anaesthetist (sugammadex group 10.6% vs neostigmine group 20% *p*=0.59). In the sugammadex group, one review was for bradycardia, while the second was for shortness of breath. Subsequent reviews for participants in the neostigmine group were for a new onset wheeze with desaturation in one patient, and for uncontrolled pain in a second patient.

### 3.6. Hospital Stay

The hospital length of stay was similar between both groups. (Group S: 4.2 (2.8–5.6) days, Group N: 5.6 (2.2–9.0) days, *p*=0.44).

### 3.7. Postoperative Follow Up

One patient was found to have received antibiotics for a chest infection on day 30 in the sugammadex group, with no patients in the neostigmine group reporting the need for antibiotics and no patients in either group reporting new or increased bronchodilator therapy in the 30 days postoperatively.  Table 3 provides a summary of all outcome data collected.

## 4. Discussion

This randomised controlled trial was constructed to assess neuromuscular blockade reversal with sugammadex compared to neostigmine in patients who were at intermediate to high risk of PPC. The trial was ceased early due to resource constraints and COVID-19. In the small numbers that were recruited there was no difference detected in the rates of postoperative pulmonary complications following reversal with sugammadex when compared with reversal with neostigmine. There was also no difference found between groups in terms of patient quality of recovery, as measured by the QoR-15 score, postoperative nausea and vomiting, a composite of PACU events and hospital length of stay, and the need for chest infection treatment in the first 30 days postoperatively. Given the small sample size before trial termination, there was no ability to assess for clinically meaningful or statistically significant differences in outcomes, and as such, the outcomes of this study cannot be directly translated into clinical care.

Despite these limitations, the experience of designing and initiating this trial has led to a number of practical recommendations for future attempts at similar endeavours. Firstly, a more clinically relevant patient-centred primary outcome would be recommended. Days Alive and at Home to Day 30 (DAH_30_), is a validated, patient-centred outcome that is associated with surgical complexity, in-hospital complications, and longer-term outcomes, which can also be easily linked to economic outcomes [21]. Additionally, good published data exists in a variety of surgical populations of mean and variability for DAH_30_ to provide for power analysis [22]. Future studies should also consider assessing a return to normal function, including normal levels of work/study, to further assess any social and economic implications of clinical outcomes/impacts.

Finally, using the QOR-15 at 30 days as a measure of quality of recovery needs an adaptation for out of hospital use, as one of the included questions is worded to enquire about “support from hospital doctors and nurses”, which is not relevant after discharge. A modified QoR-15 questionnaire to assess “receiving support from healthcare services, if you needed it” could address this incompatibility, or the use of an alternative validated quality of life metric.

We have reported the study according to randomised study standards as recommended by the Consolidated Standards of Reporting Trials (CONSORT) group [23].

## 5. Conclusions

We were not able to discern whether sugammadex compared to neostigmine was associated with a reduced rate of postoperative pulmonary complications. Future research to definitively address this question is needed and appears feasible with a well-designed and adequately funded study.

## Figures and Tables

**Table 1:** Definitions used for outcome measurements and risk stratification.

European perioperative clinical outcome (EPCO) definitions of postoperative pulmonary complications [18]	
Respiratory infection	Patient has received antibiotics for suspected respiratory infection and met one or more of the following criteria: new or changed sputum, new or changed lung opacities, fever, white blood cell count > 12 × 10^9^/litre
Respiratory failure	Postoperative PaO_2_ < 60 mm·Hg on room air, PaO_2_ : FiO_2_ ratio < 300 mm·Hg), or arterial oxyhaemoglobin saturation measured with pulse oximetry < 90% and needing oxygen therapy
Pleural effusion	Chest radiograph showing blunting of costophrenic angle, loss of sharp silhouette of ipsilateral hemidiaphragm in upright position, evidence of displacement of adjacent anatomical structures, or (in supine position) hazy opacity in one hemithorax with preserved vascular shadows
Atelectasis	Lung opacification with shift of mediastinum, hilum, or hemidiaphragm towards affected area, and compensatory over-inflation in adjacent nonatelectatic lung
Pneumothorax	Air in pleural space with no vascular bed surrounding visceral pleura

Assess respiratory risk in surgical patients in catalonia (ARISCAT) score for risk prediction of postoperative pulmonary complications [17]	
Risk factor	Score
Age 51–80	3
Age > 80	16
Preoperative SpO_2_ 91–95%	8
Preoperative SpO_2_ ≤ 90%	24
Respiratory infection in past 1 month	17
Preoperative haemoglobin < 10 gm/dl	11
Upper abdominal incision	15
Intrathoracic incision	24
Surgery duration 2-3 hours	16
Surgery duration > 3 hours	23
Emergency procedure	8
Total score-low risk: <26, intermediate risk: 26–44, high risk: ≥45	

Apfel score for risk prediction of PONV [20]	
Risk factors (1 point each) – female sex, history of ponv or motion sickness, nonsmoker, postoperative opioid treatment planned.	
Total score and risk stratification	
0	Minimal risk
1	Low risk
2	Intermediate risk
3	High risk
4	Very high risk

Definitions of postoperative care unit events	
PACU events	
(i) Any desaturation to SpO_2_ < 90%	
(ii) Need for manual airway support	
(iii) Need for oropharyngeal or nasopharyngeal airway	
(iv) Need for reintubation in PACU	
(v) Need for anaesthetist to review the patient	
(vi) Unplanned ICU admission	
PONV score	
1. No PONV	
2. PONV responsive to antiemetics	
3. PONV unresponsive to antiemetics	

PaO_2_, partial pressure of arterial oxygen; FiO_2_, fraction of inspired oxygen; SpO_2_, peripheral capillary oxygen saturation; PONV, postoperative nausea and vomiting; PACU–post anaesthesia care unit; ICU, intensive care unit.

**Table 2:** Demographic characteristics of study participants.

	Sugammadex	Neostigmine
Age (years) mean (SD)	57+/−7	58+/−7
Female sex (*n*/total)	68% (13/19)	45% (5/11)
ASA class (*n*/total)		
1	11% (2/19)	9% (1/11)
2	68% (13/19)	55% (6/11)
3	21% (4/19)	36% (4/11)
4	0% (0/19)	0% (0/11)
Height (cm)	165+/−4	171+/−6
Weight (kg)	78+/−8	81+/−18
BMI	28+/−2	28+/−5
Current smoker (*n*/total)	5% (1/19)	9% (1/11)
Duration of surgery (min)	197+/−25	204+/−51
Relaxant used (*n*/total)		
Rocuronium	21% (4/19)	45% (5/11)
Vecuronium	79% (15/19)	55% (6/11)
PONV prophylaxis (*n*/total)		
None	0% (0/19)	18% (2/11)
Dexamethasone	26% (5/19)	18% (2/11)
Ondansetron	11% (2/19)	27% (3/11)
Dexamethasone/ondansetron	58% (11/19)	36% (4/11)
Dexamethasone/droperidol	5% (1/19)	0/11 (0/11)
ARISCAT risk score (*n*/total)		
Low	33% (6/18)^*∗*^	27% (3/11)
Intermediate	67% (12/18)^*∗*^	45% (5/11)
High	0% (0/18)^*∗*^	27% (3/11)
Apfel risk score (*n*/total)		
Minimal	0% (0/19)	0% (0/11)
Low	16% (3/19)	36% (4/11)
Intermediate	74% (14/19)	64% (7/11)
High	11% (2/19)	0% (0/11)
Very high	0% (0/19)	0% (0/11)

^*∗*^one patient had no preoperative Hb, therefore could not calculate ARISCAT score. SD, standard deviation; ASA, American society of anesthesiologists; ARISCAT, assess respiratory risk in surgical patients in Catalonia, BMI, body-mass index, PONV, postoperative nausea and vomiting.

**Table 3:** Outcome data.

	Sugammadex	Neostigmine	*p*value
Postoperative pulmonary complication	0 (0–17)%	9 (0–41)%	0.37
PONV score > 1	16 (3–40)%	10 (0–44)%	0.99
Events in PACU (%)	16 (3–40)%	27 (6–60)%	0.59
QOR-15 Day 1	102 (88–116)	87 (60–104)	0.21
QoR-15 Day-30	129 (117–142)	133 (120–146)	0.61
Need for postoperative antibiotics to day 30	6 (0–29)%	0 (0–28)%	0.99
Need for new or increased bronchodilators to day 30	0 (0–19)%	0 (0–28)%	0.99
Hospital discharge summary diagnosis of respiratory infection	0 (0–18)%	0 (0–28)%	0.99
Hospital length of stay (days)	4.2 (2.8–5.6)	5.6 (2.2–9.0)	0.44

PONV, postoperative nausea and vomiting, QoR-15, quality of recovery-15 score, PACU, postanaesthetic care unit). data presented as *n* (95% CI).

## Data Availability

The datasets used and/or analysed during the current study are available from the corresponding author on reasonable request.

## Abbreviations

ARISCAT: Assess respiratory risk in surgical patients in Catalonia
ASA: American society of anesthesiologists
BMI: Body mass index
CONSORT: Consolidated standards of reporting trials
COVID: Coronavirus disease
DAH_30_: Days alive and at home to day 30
DSMC: Data and safety monitoring committee
EPCO: European perioperative clinical outcome
ICU: Intensive care unit
NHMRC: National health and medical research council
NMT: Neuromuscular twitch
NNT: Number needed to treat
PACU: Postanaesthetic care unit
PONV: postoperative nausea and vomiting
POPULAR: Postanaesthesia pulmonary complications after use of muscle relaxants
PPC: Postoperative pulmonary complication
QoR-15: Quality-of-recovery-15 score
RCT: Randomised controlled trial
STRONGER: Sugammadex versus neostigmine for reversal of neuromuscular blockade and postoperative pulmonary complications
TIVA: Total intravenous anaesthesia
TOFC: Train-of-four count.

## Supplementary Materials

Supplementary Materials Protocol 2.1 – Full Protocol of trial. CONSORT 2010 Checklist P-PERSoN–Copy of CONSORT checklist. Supplementary Materials.
