# Supplementary material for: Prevention of Postoperative Events following Reversal with Sugammadex or Neostigmine (the P-PERSoN Trial): Pilot Data Following Early Termination of a Prospective, Blinded, Randomised Trial
Source: Anesthesiol Res Pract. 2022 Jul 8;2022:4659795. doi: 10.1155/2022/4659795 (PMC9286967; doi:10.1155/2022/4659795)
Supplement: Supplementary Materials — Protocol 2.1 – Full Protocol of trial. CONSORT 2010 Checklist P-PERSoN–Copy of CONSORT checklist. Supplementary Materials. [file 4659795.f1.zip › Protocol2.1.pdf]

RESEARCH PROTOCOL

Prevention of Postperative Events following Reversal with Sugammadex or Neostigmine (P-PERSoN trial)

Version 2.1 June 2017

Universal Trials Number - U1111-1177-4335
ANZCTR Registry Number - ACTRN12616000063415
Clinical Trials.gov - NCT02825576

STATEMENT OF COMPLIANCE

This document is a protocol for a clinical research study. The study will be conducted in compliance with all stipulations of this protocol, the conditions of ethics committee approval, the NHMRC National Statement on Ethical Conduct in Human Research (2007)[1] and the Note for Guidance on Good Clinical Practice (CPMP/ICH-135/95)[2].

CONFIDENTIAL

This document is confidential and the property of the study authors.
No part of it may be transmitted, reproduced, published, or used without prior written authorisation from the study authors.

Contents

PROTOCOL SYNOPSIS 4
GLOSSARY OF ABBREVIATIONS 6
1. Study Management 6
2. INTRODUCTION AND BACKGROUND 9
3 STUDY OBJECTIVES 13
4. STUDY DESIGN 14
5. STUDY TREATMENTS 16
6. PARTICIPANT ENROLLMENT AND RANDOMISATION 16
7. STUDY VISITS AND PROCEDURES SCHEDULE 20
8. CLINICAL AND LABORATORY ASSESSMENTS 21
9. ADVERSE EVENT REPORTING 21
10. STATISTICAL METHODS 23
11. DATA MANAGEMENT 24
12. ADMINISTRATIVE ASPECTS 24
13. USE OF DATA AND PUBLICATIONS POLICY 25
14. REFERENCES 26
15. APPENDICES 29

PROTOCOL SYNOPSIS

Title	Prevention of Postperative Events following Reversal with Sugammadex or Neostigmine
Objectives	Primary:<br>• To determine if the use of sugammadex (compared with neostigmine) reduces the rate of postoperative pulmonary complications (PPCs)?<br><br>Secondary:<br>• To determine if the use of sugammadex compared with traditional reversal agents improves patient quality of recovery?<br>• To determine if the use of sugammadex compared with traditional reversal agents reduces the rate of postoperative nausea and vomiting?<br>• To determine if the use of sugammadex compared with traditional reversal agents reduces the rate of airway complications in the PACU
Study Design	Local, multi-centre, prospective, randomised, double blinded trial
Planned Sample Size	976
Selection Criteria	Eligible patients will be;<br>• >18 years old<br>• patients presenting for non-cardiac surgery and;<br>• planned operative time of over 2 hours and;<br>• plan to be intubated and to receive muscle relaxants during their anaesthetic and;<br>• plan to stay at least one night in hospital.<br><br>Additionally, patients recruited to the study will not have any of the exclusion criteria;<br>• Previous recruitment to the trial<br>• Patient refusal<br>• Weight>200kg<br>• Planned postoperative intubation and ventilation<br>• Hypersensitivity reactions to any of the study drugs<br>• Contraindications to study drugs;<br>• Mechanical obstruction of the intestinal or urinary tracts<br>• Peritonitis<br>• Liver failure with Child-Pugh class B/C<br>• Renal failure with either regular peritoneal or haemodialysis or serum creatinine >140mcgmol/L
Study Procedures	Following informed consent prior to surgery, patients will be randomised to two groups allocating drugs used for reversal of muscle relaxation;

	1. 2mg/kg sugammadex<br>2. 50mcg/kg neostigmine with 10mcg/kg glycopyrrolate<br><br>Patients will have NMT monitoring intraoperatively to ensure return of TOF count >2 prior to reversal. Muscle relaxant will be limited to rocuronium or vecuronium, at the choice of the individual anaesthetist.<br><br>As this study is planned to be a pragmatic 'real world' trial, mode of anaesthesia, analgesia, PONV prophylaxis and time of reversal will be determined by the individual treating anaesthetist,<br><br>Postoperative outcome data will be collected in the recovery unit, on postoperative day 1-3 (if still an inpatient), at hospital discharge and via a 30 day post-operative phone call.
Statistical Procedures<br>Sample Size Calculation:<br>Analysis Plan:	Conservatively estimating the baseline incidence of PPC at 7% baseline incidence and an equally conservative estimate that sugammadex can reduce this to 3% would produce a clinically relevant NNT of 29. Accepting an alpha error of 0.05 and beta error of 0.2 would require 930 patients. Allowing for 5% incomplete data and loss to follow up requires 976 patients.<br><br>Groups will be analysed on an intention-to-treat basis<br>1. PPC rate, QoR-15 score and hospital stay will be assessed as continuous variables<br>2. PONV score will be assessed as an ordinal variable<br>3. Mortality and the presence of respiratory and PACU events will be assessed as categorical variables<br><br>The effect of sugammadex on continuous variables will be analysed by 2-tailed Student T-test.<br>The effect of sugammadex on ordinal and categorical variables will be analysed by Chi-squared tests.<br>Binomial regression analysis will be performed on the categorical outcomes for the subgroup analyses.<br>Logistic regression will be performed to analyse the effect of PONV risk on PONV scores.<br><br>Appropriate statistical tests to confirm test assumptions are met will be performed. In the case of non-parametric data, the appropriate test will be performed. Interim analysis may be performed on the advice of the DSMC after 50% data completion
Duration of the study	2 years

GLOSSARY OF ABBREVIATIONS

ABBREVIATION	TERM
ARISCAT	Assess Respiratory Risk in Surgical Patients in Catalonia
ASA	American Society of Anaesthesiologists
BMI	Body mass index
GCP	Good Clinical Practice
NHMRC	National Health and Medical Research Council
NMB	Neuromuscular blocking agent
NSLHD	Northern Sydney Local Health District
PACU	Postanaesthetic care unit
PaO2	Partial pressure of oxygen in arterial blood
POD	Postoperative Day
PONV	Postoperative nausea and vomiting
PORC	Postoperative residual curarisation
PPC	Postoperative pulmonary complication
QALY	Quality Adjusted Life Year
QoR-15	Quality of recovery score (15-item)
RCT	Randomised controlled trial
SAE	Serious adverse event
SUSAR	Suspected unexpected serious adverse reaction
TOFC	Train-of-four count
TOFR	Train-of-four ratio

1. Study Management

1.1 Principal Investigator

Dr Benjamin Luke Olesnicky FANZCA, BMBS, BSc
Northern Sydney Anaesthetic Research Institute,
Department of Anaesthesia, Level 4, Royal North Shore Hospital
Reserve Rd, St Leonards, New South Wales, Australia 2065.
+61 2 94632488 . bolesnicky@hotmail.com

1.2 Associate Investigators

A/Professor Greg Knoblanche, FANZCA, MBBS
Northern Sydney Anaesthetic Research Institute,
Department of Anaesthesia, Level 4, Royal North Shore Hospital
Reserve Rd, St Leonards, New South Wales, Australia 2065.
+61 2 94632488 , gknoblanche@hotmail.com

Dr Matthew Doane
Northern Sydney Anaesthetic Research Institute,
Department of Anaesthesia, Level 4, Royal North Shore Hospital
Reserve Rd, St Leonards, New South Wales, Australia 2065.
+61 2 94632488

1.3 Statistician

External Statistical support will be provided by WriteSource Medical Pty Ltd, Lane Cove NSW 1595

1.4 Internal Trial Committees

Trial Management Committee

Dr Ben Olesnicky
Dr Matthew Doane
A/Prof Greg Knoblanche
Dr Clare Farrell

Trial Steering Committee

Dr Ben Olesnicky
Dr Matthew Doane
Dr Clare Farrell
Dr Arpit Srivastava
Dr Caroline Jackson
Dr Gerri Khong
Dr Jonothan Brock
Dr Simon Collins
Research Assistant (employment pending)

Writing Committee

Dr Ben Olesnicky
Dr Matthew Doane
A/Prof Greg Knoblanche
Dr Clare Farrell

1.5 Independent Safety and Data Monitoring Committee

Will consist of 2 anaesthetists external to the trial with input form the statistician assigned to the trial.

Data Safety and Monitoring Committee

Prof. Peter Kam
Dr Ross Wallace
Trial Statistician

1.6 Sponsor

Northern Sydney Local Health District, NSWHealth.

1.7 Proposed Budget

1.8 Funding and resources

- Northern Sydney Anaesthetic Research Institute – provision of research support and infrastructure for trial
- RNS Department Anaesthetic Funds – Department will support trial through the use of resident and registrar time for data collection and through the use of departmental infrastructure (Office Space, Computers, RedCap etc)
- Applications approved
    - MSD Investigator Grant (IIS-54809)
- Applications pending;
    - ANZCA Trials Grant

2. INTRODUCTION AND BACKGROUND

2.1 Background Information

Inadequate reversal of neuromuscular blockade is acknowledged as a common problem in anaesthetic practice. Previous work has explored both the incidence and risks associated with residual postoperative curarisation (RPOC) as well as the effectiveness of sugammadex to reverse paralytic agents and to prevent RPOC.

Residual Postoperative Curarisation – Definition

Non-depolarising muscle relaxants all work by antagonistic competition with acetylcholine for nicotinic receptors at the motor end plate. Repetitive stimulation of a motor nerve with a frequency more than 5 per second results in the release of smaller and smaller amounts of acetylcholine with each subsequent nerve action potential[3]. As a maximal twitch response needs only 20-30% nicotinic receptor activation on the muscle, in the presence of antagonism by neuromuscular blocking agents (NMB's), at occupancy levels >70%, the reduction in acetylcholine release is reflected by 'fade', whereby each of four sequential 2Hz stimulations (a train-of –four) is met with a reduced amplitude muscle contraction. This forms the basis of neuromuscular junction monitoring in anaesthesia, first outlined by researchers at Harvard medical school in 1971[4,5]. They showed that the ability to lift the head required a train-of-four ratio (TOFR, which reflects the amplitude of the fourth to first twitch amplitude) of >0.63 and later showed that the recovery of the TOFR to greater than 0.74 +/- 0.04 was associated with clinical signs including the ability to; open the eyes widely, protrude the tongue, cough and sustain head raising for at least 5 seconds[6]. Recovery of the TOFR>0.7 was also shown to be necessary to prevent clinically relevant changes in vital capacity and peak inspiratory and expiratory flows[7].

These clinical tests of function and a cut-off for TOFR of >0.7 were accepted as the target for effective reversal of neuromuscular blockade for two decades, until newer studies showed that recovery to TOFR>0.9 was needed to prevent laryngeal aspiration of contrast and dysfunction during swallowing[8,9], and that a TOFR<0.9 was associated with symptoms of residual paralysis[10]. Subsequent work has shown that depression of the hypoxic ventilatory response also occurs with TOFR<0.9[11]. Building on these previous findings, a recent systematic review of residual paralysis has recommended that the minimal acceptable level of neuromuscular recovery prior to extubation is a is a TOFR>0.9[12].

Residual Postoperative Curarisation – Incidence

A large number of studies have looked at the rate of RPOC, with a wide range of incidence rates depending on the muscle relaxant, the definition of RPOC used, the use of reversal agents and the measurement times for RPOC (time of extubation v arrival in the PACU). The lowest published reported rate for TOFR<0.9 is 13.1%, which occurred on arrival to the PACU. In this group of 84 patients, 58.3% were reversed with neostigmine[13]. The highest published rate of patients with inadequate reversal was 89%, measured on arrival to the PACU and following rocuronium use in patients aged >65[14]. No reversal was given in this group. Varying rates between these extremes have been shown in numerous other studies[15,16,17,18,19] with a 2007 meta-analysis of 3375 patients showing an overall incidence of RPOC as defined by TOFR<0.9 of 72% with long-acting NMB muscle relaxants, and 41.3% where intermediate duration NMB's were used[20]. Most concerning is the professional perception of this issue, with a survey of 2636 US and European anaesthetists in 2010 revealing that most respondents felt the rate of clinically significant residual paralysis was <1%[21].

Morbidity associated with RPOC

Residual postoperative paralysis has been linked to morbidity following surgery. The majority of morbidity has been respiratory in nature, reflecting the impairment of laryngeal reflexes and swallowing function[9,10], and the impairment of hypoxic respiratory drive at TOFR<0.9[22]. These postoperative pulmonary complications are felt to arise from an increased risk of atelectasis and micro-aspiration following inadequate reversal. A prospective study looking at 146 patients over two separate 7 day periods at Royal Perth Hospital concluded that a TOF of <0.9 was associated with increased "X-ray results consistent with postoperative atelectasis or pneumonia[23], however, only 30 patients had postoperative chest Xrays in the two cohorts.

A 2011 prospective randomised controlled trial looked at 114 orthopaedic patients randomised to neostigmine reversal or saline. The neostigmine group were extubated at TOFR = 1.0, while the saline group were extubated at loss of quantitative fade (which turned out to be TOFR 0.6-0.9). The saline group had a higher incidence of hypoxia (as defined by SpO2 <93% (51% vs 28%)[24], however, this difference was seen in the SpO2 90-93%, with no difference being seen in SpO2<90%. When using a TOFR<0.7 as a definition of residual paralysis, the incidence of postoperative hypoxia was increased in the residual paralysis group[25], as was the incidence of critical respiratory events in the PACU[26]. The same group later showed that TOFR<0.9 on arrival to the PACU was associated with an increased risk of developing hypoxia in the PACU[27,28]. A randomised controlled trial of 691 patients showed that the use of a long acting paralytic drug (pancuronium) compared with intermediate acting drugs (vecuronium and atracurium) led to an increased risk of RPOC (26% vs 5-6%) and an increased risk of pulmonary complications (16.9% vs 4.8%)[29].

The use of sugammadex to prevent RPOC

Published studies investigating the effect of sugammadex on the rate of RPOC consistently shown a reduction in RPOC incidence. Prospective data on 146 patients in Perth revealed a rate of TOFR<0.9 in 8% of patients reversed with sugammadex, compared with 53% with no reversal, and 59% in the neostigmine reversal group[23]. An observational study in a Belgian hospital showed a strong trend towards a reduction in PORC with sugammadex[30], compared with no reversal or neostigmine, while a recent randomised controlled trial in 154 general surgical patients comparing sugammadex to neostigmine/glycopyrollate showed a reduction in the rate of TOFR<0.9 from 43% to 0% with the use of sugammadex[31]. A further cohort based study showed a reduction in PORC (as defined by TOFR<0.9) from 23.9 to 4.3% using sugammadex[32].

The use of sugammadex to prevent the morbidity associated with RPOC

Although there is strong data to show the reduction in RPOC with sugammadex, the correlation to a reduction in morbidity is scant. Our previous work looked at 1257 cases, comparing cohorts before and after the introduction of routine sugammadex use. Following the introduction of routine sugammadex use, the risk of an in hospital respiratory diagnosis was decreased; OR = 0.20 (95%CI 0.05-0.72, p=0.01), while the use of sugammadex itself was associated with a reduction of in-hospital respiratory diagnoses OR = 0.26 (95%CI 0.08-0.94, p=0.04)[33].

A randomised study in 320 bariatric surgical comparing sugammadex to neostigmine showed a reduction in new postoperative pathological chest Xray changes associated with atelectasis or collapse/consolidation (16.3% vs 6.9%) with sugammadex use[34].

A retrospective investigation of postoperative outcome in 1444 patients (722 received sugammadex) analysed patients based on ASA status. The type of reversal had no effect on pulmonary outcome score between reversal groups, however there was a potentially improvement in this score with elderly ASA 3 and 4 patients. The study used a

complicated statistical analysis of the data which seemed to show a trend towards worse scores with sugammadex in younger patients, however, no further data was presented to support the paper's graphical representation[35].

Other outcome based studies comparing sugammadex with traditional reversal

Pain

An small randomised trial reported sugammadex effects on pain outcomes, reporting a clinically relevant reduction in pain scores, and reduced incidence of severe pain at 30 and 60 minutes post laparoscopic gastric banding[36].

Bleeding

Two published studies have looked at the effect of sugammadex on coagulation. In a prospective study of 1198 orthopaedic patients comparing sugammadex to neostigmine or saline, there was a transient increase in coagulation tests with sugammadex (at 10 minutes post-op)[37], but this was not associated with differences in bleeding events, thromboembolic events, transfusion rates or changes in haemoglobin. A small RCT in 50 patients undergoing sinus surgery did report a significant increase in post-op bleeding in the group randomised to sugammadex reversal[38]. This significant difference was from 2.5mL to 4.1mL of blood loss, so the clinical significance is questionable.

PONV

Four studies have looked at the impact of sugammadex on post-operative nausea and vomiting (PONV). A 2015 paper looked at PONV scores on arrival to the PACU in 100 patients having extremity surgery. They reported a statistically significant reduction in PONV scores in the sugammadex group (versus neostigmine reversal). However, both groups had a score of 0 in the reported data, and the statistical analysis was one-tailed only[39]. A randomised controlled trial in 88 patients having obesity surgery reported a reduced rate of early PONV with sugammadex compared with neostigmine (18.1-6.8%)[36]. The retrospective analysis of 1444 patients on pulmonary outcome (previously mentioned) also looked at PONV scores, demonstrating both a reduction in PONV incidence (21.5% vs 13.6%) and a reduction in the need for anti-emetic usage in the PACU (10.2% vs 13.6%) with sugammadex use[35]. Finally a retrospective case note review of 374 patients in two cohorts (before and after the introduction of routine sugammadex) found no difference in PONV incidence[40].

Haemodynamics

The effect of sugamamdex on haemodynamic parameters has also been investigated with one paper showing traditional reversal resulting in a higher mean arterial pressure and heart rate compared with sugammadex in paediatric neurosurgical patients up to 10 minutes post reversal[41]. A separate study in 100 ASA 1 and 2 patients showed a reduction in heart rate with traditional reversal up to 24 hours postoperatively[39].

Patient Experience

An observational study in 101 patients looked at the effect of sugammadex on postoperative quality of recovery scores. They showed an improvement in physiological and noiceceptive domains of the quality of recovery score at 40 minutes, and higher patient satisfaction rates in the sugammadex group[42].

Other

A small randomised trial comparing sugammadex and traditional reversal showed a marked reduction in the incidence of dry mouth postoperatively[43].

2.2 Research Question

1. Does the use of sugammadex compared with neostigmine reduce the rate of postoperative pulmonary complications (PPCs)?
2. Does the use of sugammadex compared with traditional reversal agents improve patient quality of recovery?
3. Does the use of sugammadex compared with traditional reversal agents reduce the rate of postoperative nausea and vomiting?
4. Does the use of sugammadex compared with traditional reversal agents reduce the rate of airway complications in the PACU?

2.3 Rationale for Current Study

Whether or not sugammadex is superior to neostigmine based techniques in terms of prevention of the morbidity associated with residual paralysis have never been shown in a prospective randomised trial. P-PERSoN is a prospective multi-centre, double blinded, randomised controlled trial to compare neostigmine/glycopyrrolate and sugammadex reversal. The primary outcome is in-hospital respiratory events. Secondary outcomes will be; recovery room airway and desaturation events, hospital stay, quality of recovery scores and 30-day respiratory morbidity.

3 STUDY OBJECTIVES

3.1 Primary Objective

- To determine if the use of sugammadex (compared with neostigmine) reduces the rate of postoperative pulmonary complications (PPCs)? The primary study hypothesis is that sugammadex will reduce the rate of PPCs.

3.2 Secondary Objectives

- To determine if the use of sugammadex compared with traditional reversal agents improves patient quality of recovery?
- To determine if the use of sugammadex compared with traditional reversal agents reduces the rate of postoperative nausea and vomiting?
- To determine if the use of sugammadex compared with traditional reversal agents reduces the rate of airway complications in the PACU

4. STUDY DESIGN

4.1 Type of Study

Multi-centre, prospective, randomised, double-blinded trial

4.2 Study Design

Following recruitment through a preoperative consult (outside of the theatre complex, either in preadmission clinic or the preoperative ward for same day admission patients), patients will be given written information regarding the study and consented after having adequate time to read the PIS. On arrival to the operating theatre complex, written consent will be confirmed by the treating anaesthetist. Baseline data will then be collected.

Following induction, patients will be allocated to one of two groups;

1. Sugammadex Reversal Group
2. Neostigmine Reversal Group

A second anaesthetist will draw up a standardised dose of reversal agent based on group allocation and deliver this to the treating anaesthetist. The only labelling of the syringe will be 'reversal' and the patient study number. The second anaesthetist will not be involved in the management of the trial patient other than drawing up of the study drug. The treating anaesthetist will be blinded to the patient group.

The reversal dose will be standardised to 10mL, with 1mL/10kg reflecting the recommended reversal dose. Two 10mL syringes can be used for patients >100kg.

The reversal in both groups is of the following solutions;

1. Sugammadex group – 200mg in 10mL = 2mg/kg dose
2. Neostigmine/glycopyrrolate group – 5mg neostigmine and 1mg glycopyrrolate in 10 mL= 50mcg/kg neostigmine and 10mcg/kg glycopyrrolate.

Patients will have NMT monitoring intraoperatively to ensure return of TOFC>2 prior to reversal. Muscle relaxant will be limited to rocuronium or vecuronium, at the choice of the individual anaesthetist.

As this study is planned to be a 'real world' trial, mode of anaesthesia, analgesia, PONV prophylaxis and time of reversal will be determined by the individual treating anaesthetist, Data analysis will be performed to identify if differences in these factors occur between both groups with an impact analysis of these differences, if they occur. There will be no interventions prohibited during the trial.

Postoperative data will be collected in the recovery unit, on postoperative day 1 and 2 (if still an inpatient), at hospital discharge and at a 30 day post-operative phone call. Collected data is shown in appendix A.

4.3 Number of Participants

972 (Nine hundred and seventy-two)

4.4 Study sites

Currently, the trial is planned to be conducted at metropolitan public hospitals in Sydney, Australia (Royal North Shore Hospital, North Shore Private Hospital, Westmead Hospital, Hornsby Hospital, Nepean Hospital and Mona Vale Hospital). Depending on the ability to meet recruitment targets of greater than 20 patients per week, additional hospitals in Sydney and Australia may be recruited. All additional hospitals will be subject to the approval of an alteration of the ethics approval and local site governance.

4.5 Expected Duration of Study

Utilizing four centres, and estimating a recruitment of 20 patients per week into the study once underway, we estimate completion of the data collection within 18 months. Additional recruitment centres may reduce the time taken even further. Analysis of the data and presentation of the results are expected to take another 6 months, giving an estimate of 2 years from commencement of the study until the outcomes are fully appreciated and revealed.

4.6 Primary and Secondary Outcome Measures

Our primary outcome will be the incidence of PPC's as defined by the ARISCAT (The Assess Respiratory Risk in Surgical Patients in Catalonia) group[46] (defined in Appendix 1). Secondary outcomes will look at quality of recovery scores utilising a validated measurement tool (the QoR-15)[45], the incidence of PONV and the need for anti-emetics in recovery, and the incidence of a defined set of airway events in recovery. Subgroup analysis will look at groups stratified to different risks of PPC (as defined by the ARISCAT score[46]), risk of PONV (as defined by the Apfel score[47]) patients with TIVA vs volatile based anaesthesia, patient BMI and ASA grade of the patient. If a difference between groups occurs, QoR-15 scores will be converted to QALYs to provide an economic analysis of any benefits of sugammadex. Any postoperative chest xrays will be reviewed by a blinded radiologist with reference to the ARISCAT criteria.

Day 1-3 data will be collected by research personnel (nursing staff, research assistants, SRMOs and registrars) depending on the process established at each hospital for data collection. The discharge review and 30 day follow up phone call will be performed by the research personnel at Royal North Shore hospital.

5. STUDY TREATMENTS

5.1 Treatment Arms

5.1.1 Description

The two groups will be defined as the sugammadex group and the neostigmine group

5.1.2 Dosage and Route of Administration

The reversal dose will be given intravenously and standardised to 10mL, with 1mL/10kg reflecting the recommended reversal dose. Two 10mL syringes can be used for patients 100kg -200kg.

The reversal in both groups is as follows;

- Sugammadex group – 200mg in 10mL = 2mg/kg dose
- Neostigmine group – 5mg neostigmine and 1mg glycopyrrolate in 10 mL= 50mcg/kg neostigmine and 10mcg/kg glycopyrrolate**.**

5.1.3 Dose modification

There is no need for dose modification for patients, as those with renal or hepatic disease will be excluded from the study

5.2 Preparation and administration of study drug

A second person will draw up a standardised dose of reversal agent based on group allocation and deliver this to the treating anaesthetist. The only labelling of the syringe will be 'reversal' and the patient study number. The second person will not be involved in the management of the trial patient other than drawing up of the study drug and delivering it to the treating anaesthetist. The treating anaesthetist will be blinded to the patient group.

5.3 Measurement of participant compliance

The study data sheets will include a confirmation tick box that the correct dose of study drug was given to the study patient.

5.4 Excluded medications and treatments

As this study is planned to be a 'real world' trial, mode of anaesthesia, analgesia, PONV prophylaxis and time of reversal will be determined by the individual treating anaesthetist,. Analysis of data will be performed to identify if differences in these factors occur between both groups, with impact analysis of the differences if they occur. There will be no interventions prohibited during the trial

6. PARTICIPANT ENROLLMENT AND RANDOMISATION

6.1 Recruitment

Recruitment will be through during a preoperative consult at each hospital site. Patients that meet the criteria for study inclusion will be approached by study personnel during their routine pre-anaesthetic consultation and the study discussed with the patient. At this stage, patients will be given written information regarding the study for reading in

their own time (see appendix) and written, signed consent obtained. Patients will only be initially approached outside of the theatre complex, with ample time (at least one hour) between giving written information and formal consent occurring. On the day of their operation, patients will be again offered a chance to answer questions regarding the study prior to written informed consent being confirmed by an anaesthetist. Each site investigator will be responsible for recruitment at their site.

6.2 Eligibility Criteria

6.2.1 Inclusion Criteria

- Greater than 18 years old and;
- presenting for non-cardiac surgery and;
- planned operative time of over 2 hours and;
- plan to be intubated and to receive muscle relaxants during their anaesthetic and;
- plan to stay at least one night in hospital.

.

6.2.2 Exclusion Criteria

- Previous recruitment to the trial
- Patient refusal
- Cognitive Imparment, or language proficiency leading to inability to complete QoR-15 questionnaire.
- Weight>200kg
- Planned postoperative intubation and ventilation
- Contraindications to sugammadex
    - Hypersensitivity reactions to sugammadex
- Contraindications to neostigmine
    - Hypersensitivity to neostigmine
    - Mechanical obstruction of the intestinal or urinary tracts
    - Peritonitis
- Contraindications to aminosteroid NMB's (rocuronium/vecuronium)
    - Hypersensitivity reactions to rocuronium/vecuronium or the bromide ion
- Liver failure with Child-Pugh class B/C
- Renal failure with either regular peritoneal or haemodialysis or serum creatinine >140mcgmol/L
- Women lactating, pregnant or of childbearing potential who are not willing to avoid pregnancy during the study

6.3 Informed Consent Process

Informed written consent will be obtained prior to the theatre complex visit. The person consenting will confirm with the patient;

- the study has been adequately explained to them
- they have received written information regarding the study
- they are comfortable they have had adequate time to read the written information
- they were happy that they understood the written information

Any further questions about the study will be answered at this time and the patient will be asked to sign the study consent form. The consent form will be kept with the patient data sheets.

6.4 Enrolment and Randomisation Procedures

The participant will be enrolled into the study after the informed consent process has been completed and all inclusion and exclusion criteria have been met. The participant will receive a study enrolment number and this will be documented in the participant's medical record and on all study documents.

Randomisation will be via computer based simple randomisation software (www.randomizer.org), with a sequential study number of 001 through 976 allocated to one of the two groups. This group allocation will then be placed in an opaque envelope (with the study number only on the outside). Following induction, a second anaesthetist will select the next sequentially labelled envelope which will allocate the patient to one of the to groups.

Batches of sequentially numbered envelopes will also be transported to each site by trial personnel. Each site will have enough envelopes to ensure supply relative to their trial recruitment activity.

6.5 Blinding Arrangements

The second anaesthetist (who selects the envelope) will not be involved in the management of the trial patient other than drawing up of the study drug will only be labelled with 'reversal' and the patient study number. The allocation group inside the envelope will have instructions for drawing up of the reversal drugs for the second anaesthetist and a reminder to ensure the drug is drawn up in a separate room to the treating anaesthetist and that the group allocation is kept blinded from all but the second anaesthetist.

6.6 Breaking of the Study Blind

The envelope will be resealed with a patient sticker and kept with the patient notes for any situation where unblinding of the allocation is needed rapidly in the operating theatre. The study number will also be recorded on the study data sheets and in the inpatient notes to allow for unblinding from a master copy of allocations that is kept securely by the study investigators. Unblinding will be completely at the request of the treating physician if they feel it is clinically necessary. Any unblinding events will be recorded and the reason for this reported. Unblinding events will be outlined in the final study report.

6.7 Participant Withdrawal

Clinical study participants will be advised that:

- They may voluntarily withdraw from the clinical study at any time
- They may voluntarily withdraw from the clinical study for any reason
- They are not obligated to reveal the reasons for withdrawing from the study to the Principal Investigator or Co- Investigator.
- Their decision to withdraw from the study will not affect their medical care.

Where a clinical study participant withdraws from a study, the principal investigator or co-investigator(s) may be interested in the reason(s) for voluntary withdrawal from the study. In such cases the Principal Investigator or Co-Investigator(s) may enquire why the clinical study participant has withdrawn from the study. Clinical study participants who choose to withdraw from the study will be advised to remain under the care of an appropriately experienced clinician.

It is also expected that a percentage of clinical study participants will not return for follow up at the required intervals. Such cases will be reported with the study results.

In all cases where a clinical study participant withdraws from a study, the last known clinical status of the clinical study participant will be reported with the study results.

The principal investigator or co-investigator may withdraw a clinical study participant from a study. In such cases the reason(s) for withdrawing a clinical study participant from the study will be documented in the study results and the patient's medical notes.

Participants who withdraw from the clinical study will not need to be replaced as the initial recruitment target of 976 patients makes concessions for this occurring.

7. STUDY VISITS AND PROCEDURES SCHEDULE

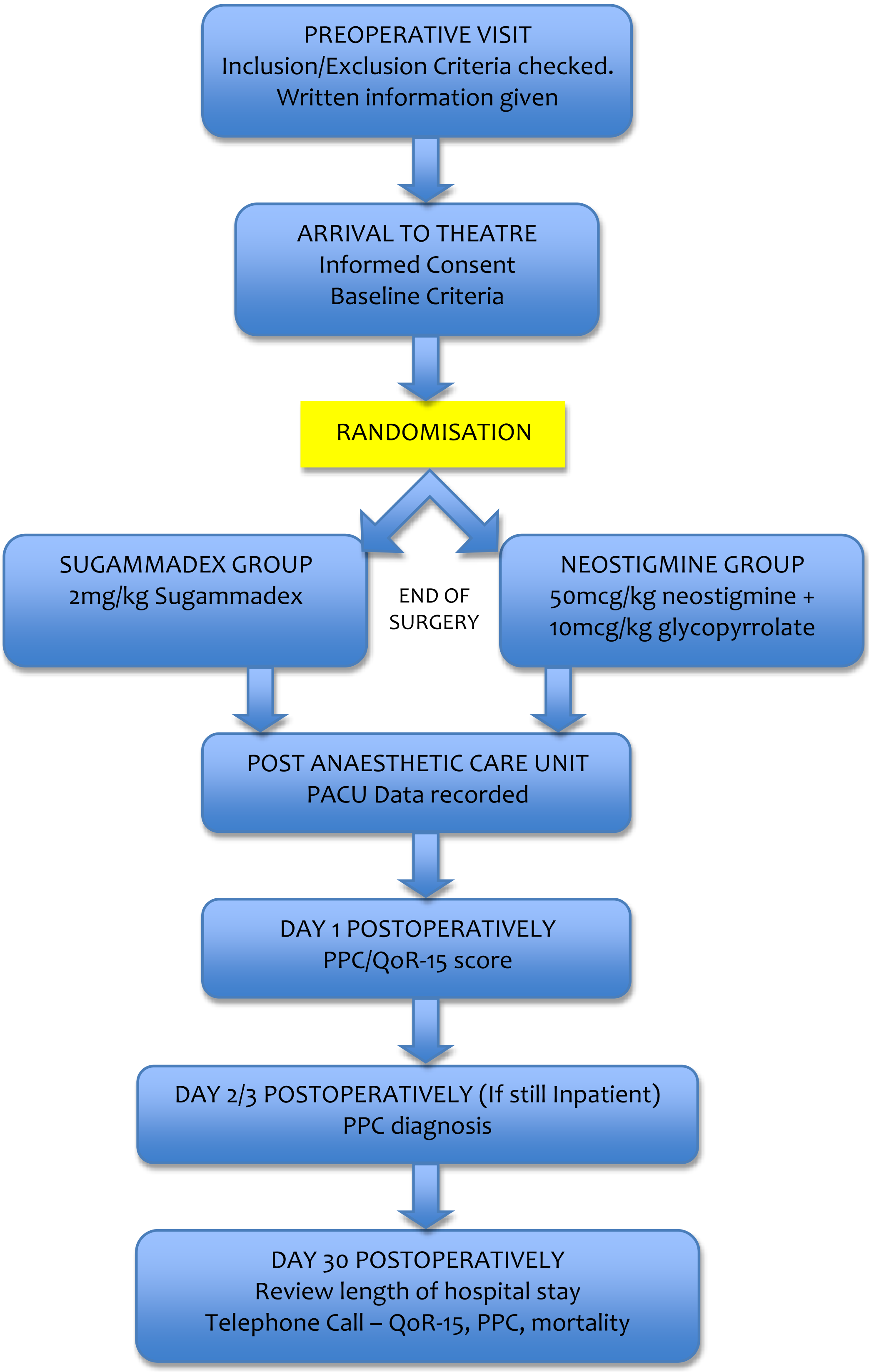

8. CLINICAL AND LABORATORY ASSESSMENTS

There are no specific clinical or laboratory studies required for this study

9. ADVERSE EVENT REPORTING

Adverse events will be managed as per the requirements of the National Health and Medical Research Council, Australian Health Ethics Committee (AHEC) Position Statement "*Monitoring and reporting of safety for clinical trials involving therapeutic products*" (May 2009)

9.1 Definitions

Adverse event

An adverse event for medications is also referred to as an adverse experience, which is any untoward medical occurrence in a patient or clinical investigation participant administered a pharmaceutical product and which does not necessarily have a causal relationship with this treatment. Adverse events can be suspected (in keeping with the approved product information for the drug) or unexpected (nature or severity is not consistent with the applicable scientific drug information)

An adverse event can therefore be any unfavourable and unintended sign, symptom, or disease temporally associated with the use of a medicinal (investigational) product, whether or not related to the medicinal (investigational) product. Adverse events can further be classified as mild, moderate or severe

- Mild - causes minimal discomfort to the patient and not interfering with activities of daily living.
- Moderate – interferes with the patient's normal everyday activities.
- Severe - prevents normal everyday activities and/or requires therapeutic intervention (i.e. use of a prescription drug or hospitalisation), but is not usually life-threatening.

9.2 Assessment and Documentation of Adverse Events

Any adverse events will be documented in the patient's study data forms on the day they are identified. On identification of an adverse event, the investigator/clinical staff will document;

- Nature of the reaction
- Time of onset after surgery
- Duration
- Severity
- Any corrective treatment given

The DSMC will regularly review any documented adverse events and report directly to the investigators and HREC if they feel that a review is needed.

9.3 Eliciting Adverse Event Information

All adverse events experienced by a patient that is either observed by the investigator, by one of the clinical staff, reported by the patient spontaneously or in response to a direct question will be recorded in the patient data forms. Data on specific adverse events will also be collected from the discharge summaries on review (MI, CVA, anaphylaxis, Renal Failure).

9.3.1 SAEs

Serious adverse event (SAE):
An unforeseen medical event that occurs in the course of clinical research that:

- results in participant death
- is life-threatening to the participant
- requires the inpatient hospitalisation or prolongation of existing hospitalisation for the participant leads to the participant having a persistent or significant disability/incapacity.

For medicines, also referred to as serious adverse drug reaction, any untoward medical occurrence that at any dose:

- results in death;
- is life-threatening;
- requires in-patient hospitalisation or prolongation of existing hospitalisation;
- results in persistent or significant disability/incapacity;
- is a congenital anomaly/birth defect; or
- is a medically important event or reaction.

The term 'life-threatening' in the definition of 'serious' refers to an event in which the patient was at risk of death at the time of the event; it does not refer to an event/reaction which hypothetically might have caused death if it were more severe.

9.3.2 SUSARs

Suspected Unexpected Serious Adverse Reaction (SUSAR) are defined as;

- Any adverse events that are suspected to be related to an investigational medicinal product and that are both unexpected and serious.
- A serious adverse event for which there is some degree of probability that the event is an adverse reaction to the administered drug and the adverse reaction is unexpected.
- A serious event NOT outlined in the study protocol or information sheet.

All SAEs/SUSARs will be reported directly to the Northern Sydney Local Health District HREC;

- Using the approved SAE/SUSAR reporting form, available at
  http://www.nslhd.health.nsw.gov.au/AboutUs/Research/Office/Pages/Standard-Forms.aspx
- Within 24 hours of the researchers becoming aware of the event

Serious adverse events in Australia will also be reported to the drug safety and evaluation branch of the Therapeutic Goods Association through their website; https://www.tga.gov.au/reporting-medicine-and-vaccine-adverse-events-0. The investigators will ensure a TGA report is completed within 7 days of a SAE or SUSAR occurring and that any SAE or SUSAR is reported to the DSMC.

10. STATISTICAL METHODS

10.1 Sample Size Estimation

Previous studies have shown a baseline incidence of postoperative pulmonary complications (PPC's) between 2-39%[48]. The largest prospective study looking at a defined set of PPC's showed an overall incidence of 7.9%, stratified into low risk (3.4%), intermediate risk (13.0%) and high risk (38.0%)[46]. Based on a conservative estimate of 7% baseline incidence and a conservative estimate that sugammadex can reduce this to 3% (based on our retrospective pilot data showing an relative risk reduction of 0.21, which would reduce the incidence to 1.47%) would produce a clinically relevant NNT of 29. Accepting an alpha error of 0.05 and beta error of 0.2 would require 930 patients[49]. Allowing for 5% incomplete data and loss to follow up requires 976 patients.

10.2 Statistical Analysis Plan

Statistical analysis will be performed based on the outcome.
1. PPC rate, QoR-15 score, hospital stay will be assessed as continuous variables
2. PONV score will be assessed as an ordinal variable
3. Mortality and the presence of respiratory and PACU events will be assessed as categorical variables

The effect of sugammadex on continuous variables will be analysed by 2-tailed Student T-test
The effect of sugammadex on ordinal and categorical variables will be analysed by Chi-squared tests
Binomial regression analysis will be performed on the categorical outcomes for the subgroup analyses
Logistic regression will be performed to analyse the effect of PONV risk on PONV scores.

Statistical analysis appropriate to confirm test assumptions are met will be performed.

Compliance with study protocol will be assessed and recorded in the study results and groups will be analysed on an intention-to-treat basis.

10.3 Interim Analyses

An interim-analysis may be performed on the primary endpoint when 488 (50% of patients) have been randomised and have completed the 30 day follow-up. This will be performed on the advice of the DSMC. The DSMC will have unblinded access to all data and will discuss the results of the interim-analysis with the management committee in a joint meeting following interim analysis. The management committee decides on the continuation of the trial and will report to the central ethics committee. The Haybittle-Peto approach is used where the trial will be ended using symmetric stopping boundaries at $P < 0.001$. The trial will not be stopped in case of futility of treatment effect, unless the DSMC during the course of safety monitoring advises otherwise. In this case DSMC will discuss potential stopping for futility with the management committee.

At this time, a decision will be made by the DSMC if recruitment needs to be changed in view of a low event rate and inadequate power with trial continuation.

11. DATA MANAGEMENT

11.1 Data Collection

Data will be directly entered onto patient data sheets by clinical staff. They will record data as required by the study protocol at each step. Data sheets are also designed to document compliance with the study protocol. Any reasons for non compliance will also be documented and reviewed by the DSMC.

Data from paper data forms will be entered into a spreadsheet by the study investigators. At the completion of data entry, a small sample of 30 patients will be assessed for accuracy of data entry by directly comparing the data entry sheets and the spreadsheet by two independent observers. Error rates> 0.5% (of all data points for those 30 patients) in data transfer will trigger a complete review of all data sheets and all 976 participants will be directly double checked by two separate individuals.

11.2 Data Storage

Once completed, data sheets will be transferred to Royal North Shore hospital by site investigators. They will then be stored in a locked drawer within the Department of Anaesthesia at Royal North Shore Hospital. Data from these source documents will also be entered into a strong password protected spreadsheet hosted on a password protected hospital network computer within the anaesthetic department.

11.3 Data Confidentiality

The source data collection sheets will be kept in a locked office at the Department of Anaesthesia, Royal North Shore Hospital (itself only accessable through keycard access and monitored by CCTV). The source documents will be the only documents containing patient name and contact details. Data storage of the spreadsheet is compliant with Australian Privacy Principles for the storage of medical data. All data in the spreadsheet will be deidentified, containing only age and sex, together with the study idenfication code (re-identifiable data). Re-identifiable data may need to be stored on the personal computers on the study investigators an/or the external biostatistician to allow for analysis of trial data and presentation of the results for presentation or publication

11.4 Study Record Retention

Study data will be kept for a minimum of 15 years, consistent with NSLHD data retention policy

12. ADMINISTRATIVE ASPECTS

The P-Person trial has been registered with the ANZCTR and with clinicaltrials.gov.

12.1 Independent HREC approval

This study has been submitted to the Northern Sydney Local Health District HREC.

12.2 Amendments to the protocol

Any amendments will be submitted to the HREC for review prior to implementation as per HREC guidelines.

12.3 Protocol deviations

Any protocol deviations will be submitted to the HREC for review.

12.4 Participant reimbursement

There are no reimbursements to individual participants for participation in the study. Dependant on grant application success, there may be reimbursement to sites for patient recruitments on a per patient recruitment stipend.

12.5 Financial disclosure and conflicts of interest

We have been accepted for an MSD grant (ethics approval dependent) for the supply of the sugammadex for the trial. MSD distribute sugammadex as Bridion™ in Australia and New Zealand.

Acceptance of a grant from MSD will be under the strict understanding that MSD have no involvement whatsoever in the trial protocol, trial process or analysis of data, nor do they have access to the raw trial data. MSD also have no involvement in any manuscript production or revision, nor do they have any control over the publication of any data from this study.

13. USE OF DATA AND PUBLICATIONS POLICY

The data will be analysed and disseminated through published scientific articles. The aim would be to have publication in a major anaesthetic journal. Findings will also be available through the ANZCTR website and will be prepared for presentations at appropriate anaesthetic scientific meetings.

The authorship of the study is planned to include the writing committee of the study, with acknowledgements to site investigators and external statisticians. Changes to the authorship may occur, pending the unanimous approval of the writing committee.

14. REFERENCES

1 National Statement on Ethical Conduct in Human Research 2007 (Updated May 2015). The National Health and Medical Research Council, the Australian Research Council and the Australian Vice-Chancellors' Committee. Commonwealth of Australia, Canberra.

2 ICH Topic E6 – Guideline for Good Clinical Practice – Note for Guidance on Good Clinical Practice - Annotated with TGA comments(CPMP/ICH/135/95).

3 Boon, A. J. "Assessing The Neuromuscular Junction With Repetitive Stimulation Studies." Clinical Neurophysiology. Vol. 3. New York: Oxford UP, 2009. 369-84. Print

4 Ali HH, Utting JE, Gray TC: Quantitative assessment of residual antidepolarizing block (part I). Brit J Anaesth 43:473-477, 1971

5 Ali HH, Utting JE, Gray TC: Quantitative assessment of residual antidepolarizing block (part II). Brit J Anaesth 43:478-485, 1971

6 Ali HH, Kitz RJ. Evaluation of recovery from nondepolarizing neuromuscular block, using a digital neuromuscular transmission analyzer: preliminary report. Anesth Analg 1973;52:740–5

7 Ali HH, Wilson RS, Savarese JJ, Kitz RJ: The effects of tubocurarine on indirect elicited train-of-four muscle responses and respiratory measurements in humans. Br J Anaesth 1975; 47:570–4

8 Eriksson LI, Sundman E, Olsson R, Nilsson L, Witt H, Ekberg O, Kuylenstierna R. Functional assessment of the pharynx at rest and during swallowing in partially paralyzed humans: simultaneous videomanometry and mechanomyography of awake human volunteers. Anesthesiology 1997;87:1035– 43

9 Sundman E, Witt H, Olsson R, Ekberg O, Kuylenstierna R, Eriksson LI. The incidence and mechanisms of pharyngeal and upper esophageal dysfunction in partially paralyzed humans. Pharyngeal videoradiography and simultaneous manometry after atracurium. Anesthesiology 2000;92:977– 84

10 Kopman AF, Yee PS, Neuman GG. Relationship of the train- of-four fade ratio to clinical signs and symptoms of residual paralysis in awake volunteers. Anesthesiology 1997;86:765–71

11 Eriksson LI. Reduced hypoxic chemosensitivity in partially paralysed man. A new property of muscle relaxants? Acta Anaesthesiol Scand 1996;40:520 –3

12 Murphy GS, Brull SJ. Residual neuromuscular block: lessons unlearned. Part I: definitions, incidence, and adverse physiologic effects of residual neuromuscular block. Anesth Analg. 2010 Jul;111(1):120-8.

13 Kaan N, Kocaturk O, Kurt I, Cicek H. The incidence of residual neuromuscular blockade associated with single dose of intermediate-acting neuromuscular blocking drugs. N Middle East J Anaesthesiol. 2012 Feb;21(4):535-41

14 Pietraszewski P, Gaszyński T. Residual neuromuscular block in elderly patients after surgical procedures under general anaesthesia with rocuronium. Anaesthesiol Intensive Ther. 2013 Apr-Jun;45(2):77-81.

15 Maybauer DM, Geldner G, Blobner M, Pühringer F, Hofmockel R, Rex C, Wulf HF, Eberhart L, Arndt C, Eikermann M. Incidence and duration of residual paralysis at the end of surgery after multiple administrations of cisatracurium and rocuronium.Anaesthesia. 2007 Jan;62(1):12-7.

16 Motamed C Bourgain JL D'Hollander A. Survey of muscle relaxant effects management with a kinemyographic-based data archiving system: a retrospective quantitative and contextual quality control approach. Journal of clinical monitoring and computing. 2013 Dec 1;27(6):669-76.

17 Thilen SR, Hansen BE, Ramaiah R, Kent CD, Treggiari MM, Bhananker SM. Intraoperative neuromuscular monitoring site and residual paralysis. Survey of Anesthesiology. 2013 Jun 1;57(3):156-7.Debaene B et al Anesthesiology 2003 May;98(5):1042-8

18 Kim KS, Lew SH, Cho HY, Cheong MA. Residual paralysis induced by either vecuronium or rocuronium after reversal with pyridostigmine. Anesthesia & Analgesia. 2002 Dec 1;95(6):1656-60.Gatke MR et al Acta Anasthesiol Scand 2002 Feb;46(2):207-13

19 Baillard C, Gehan G, Reboul-Marty J, Larmignat P, Samama CM, Cupa M. Residual curarization in the recovery room after vecuronium. British journal of anaesthesia. 2000 Mar 1;84(3):394-5.Bissinger U et al Physiol Res. 2000;49(4):455-62.

20 Naguib M, Kopman AF, Ensor JE. Neuromuscular monitoring and postoperative residual curarisation: a meta-analysis. British journal of anaesthesia. 2007 Mar 1;98(3):302-16.

21 Naguib, M, Kopman, A. A Survey of Current Management of Neuromuscular Block in the United States and Europe. Anesth Analg 2010 July;111(1) 110-119.

22 Eriksson LI. Reduced hypoxic chemosensitivity in partially paralysed man. A new property of muscle relaxants? Acta Anaesthesiol Scand 1996;40:520 –3

23 Ledowski T, Hillyard S, O'Dea B, Archer R, Vilas-Boas F, Kyle B. Introduction of sugammadex as standard reversal agent: Impact on the incidence of residual neuromuscular blockade and postoperative patient outcome. Indian J Anaesth 2013;57:46-51

24 Sauer M, Stahn A, Soltesz S, Noeldge-Schomburg G, Mencke T. The influence of residual neuromuscular block on the incidence of critical respiratory events. A randomised, prospective, placebo-controlled trial. European Journal of Anaesthesiology (EJA). 2011 Dec 1;28(12):842-8.

25 Bissinger U, Schimek F, Lenz G. Postoperative residual paralysis and respiratory status: a comparative study of pancuronium and vecuronium. Physiological Research. 2000 Jan 1;49(4):455-62

26 Murphy GS, Szokol JW, Marymont JH, Greenberg SB, Avram MJ, Vender JS. Residual neuromuscular blockade and critical respiratory events in the postanesthesia care unit. Anesth Analg 2008;107:130–7

27 Murphy GS, Szokol JW, Franklin M, Marymont JH, Avram MJ, Vender JS. Postanesthesia care unit recovery times and neuro- muscular blocking drugs: a prospective study of orthopedic surgical patients randomized to receive pancuronium or rocu- ronium. Anesth Analg 2004;98:193–200.

28 Murphy GS, Szokol JW, Marymont JH, Greenberg SB, Avram MJ, Vender JS, Nisman M. Intraoperative acceleromyographic monitoring reduces the risk of residual neuromuscular blockade and adverse respiratory events in the postanesthesia care unit. Anesthesiology 2008;109:389–98

29 Berg H, Roed J, Viby-Mogensen J, Mortensen CR, Engbaek J, Skovgaard LT, Krintel JJ. Residual neuromuscular block is a risk factor for postoperative pulmonary complications. A pro- spective, randomized, and blinded study of postoperative pulmonary complications after atracurium, vecuronium and pancuronium. Acta Anaesthesiol Scand 1997;41:1095–103

30 Cammu, G. V., Smet, V., De Jongh, K., Vandeput, D. A prospective, observational study comparing postoperative residual curarisation and early adverse respiratory events in patients reversed with neostigmine or sugammadex or after apparent spontaneous recovery. Anaesth Intens Care, 2012;40(6):999.

31 Brueckmann B, Sasaki N, Grobara p, Li MK, Woo T, de Bie J, Maktabi M, Lee J, Kwo J, Pino R, Sabouri AS, McGovern F, Staehr-Rye AK, Eikermann M. Effects of sugammadex on incidence of postoperative residual neuromuscular blockade: a randomized, controlled study. . Br. J. Anaesth. 2015;115 (5): 743-751.

32 Kotake Y, Ochiai R, Suzuki T, Ogawa S, Takagi S, Ozaki M, Nakatsuka I, Takeda J. Reversal with sugammadex in the absence of monitoring did not preclude residual neuromuscular block. Anesth Analg 2013;117(2):345-351.

33 Olesnicky BL, Traill C, Marroquin-Harris BF. The effect of routine sugammadex use on postoperative respiratory events. Unpublished Data on file.

34 Llauradó S, Sabaté A, Ferreres E, Camprubí I, Cabrera A. Postoperative respiratory outcomes in laparoscopic bariatric surgery: comparison of a prospective group of patients whose neuromuscular blockade was reverted with sugammadex and a historical one reverted with neostigmine. Rev Esp Anestesiol Reanim. 2014 Dec;61(10):565-70.

35 Ledowski T, Falke L, Johnston F, Gillies E, Greenaway M, De Mel A, Tiong WS, Phillips M.Retrospective investigation of postoperative outcome after reversal of residual neuromuscular blockade: sugammadex, neostigmine or no reversal Eur J Anaesthesiol. 2014 Aug;31(8):423-9.

36 Castro Jr DS, Leão P, Borges S, Gomes L, Pacheco M, Figueiredo P. Sugammadex Reduces Postoperative Pain After Laparoscopic Bariatric Surgery: A Randomized Trial. Surgical Laparoscopy Endoscopy & Percutaneous Techniques. 2014 Oct 1;24(5):420-3.

37 Rahe-Meyer N, Fennema H, Schulman S, Klimscha W, Przemeck M, Blobner M, Wulf H, Speek M, McCrary SC, Williams-Herman D, Woo T. Effect of Reversal of Neuromuscular Blockade with Sugammadex versus Usual Care on Bleeding Risk in a Randomized Study of Surgical Patients. Anesthesiology. 2014 Nov 1;121(5):969-77.

38 Taş N, Korkmaz H, Yağan Ö, Korkmaz M. Effect of Sugammadex on Postoperative Bleeding and Coagulation Parameters After Septoplasty: A Randomized Prospective Study. Medical science monitor: international medical

journal of experimental and clinical research. 2015;21:2382.Ghoneim AA et al Saudi J Anaesth. 2015 Jul-Sep;9(3):247-52

39 Koyuncu, O. Turhanoglu, S. Akkurt, CO. Karcıoglu M. Ozkan M., Ozer C. Turan, A. Comparison of sugammadex and conventional reversal on postoperative nausea and vomiting: a randomized, blinded trial. Journal of Clinical Anesthesia, 2015: 27(1), 51-56.

40 Watts RW, London JA, van Wijk RM, Lui YL. The influence of unrestricted use of sugammadex on clinical anaesthetic practice in a tertiary teaching hospital. Anaesthesia and intensive care. 2012 Mar 1;40(2):333.

41 Ghoneim AA, El Beltagy MA. Comparative study between sugammadex and neostigmine in neurosurgical anesthesia in pediatric patients. Saudi J Anaesth. 2015 Jul-Sep; 9(3): 247–252.

42 Amorim P, Lagarto F, Gomes B, Esteves S, Bismarck J, Rodrigues N, Nogueira M. Neostigmine vs. sugammadex: observational cohort study comparing the quality of recovery using the Postoperative Quality Recovery Scale. Acta Anaesthesiologica Scandinavica. 2014 Oct 1;58(9):1101-10

43 Sacan O, White PF, Tufanogullari B, Klein K. Sugammadex reversal of rocuronium-induced neuromuscular blockade: a comparison with neostigmine–glycopyrrolate and edrophonium–atropine. Anesthesia & Analgesia. 2007 Mar 1;104(3):569-74.

44 Canet J, Sabaté S, Mazo V, Gallart L, de Abreu MG, Belda J, Langeron O, Hoeft A, Pelosi P; PERISCOPE group. Development and validation of a score to predict postoperative respiratory failure in a multicentre European cohort: A prospective, observational study. Eur J Anaesthesiol. 2015 Jul;32(7):458-70.

45 Stark PA, Myles PS, Burke JA. Development and Psychometric Evaluation of a Postoperative Quality of Recovery ScoreThe QoR-15. Anaesthesiology 2013;118(6):1332-40.

46 Mazo V1, Sabaté S, Canet J, Gallart L, de Abreu MG, Belda J, Langeron O, Hoeft A, Pelosi P. Prospective external validation of a predictive score for postoperative pulmonary complications. Anesthesiol. 2014; 121(2):219-31.

47 Apfel CC, Läärä E, Koivuranta M, Greim CA, Roewer N. A simplified risk score for predicting postoperative nausea and vomiting: conclusions from cross-validations between two centers. Anesthesiology 1999;91:693–700.

48 Bapoje, S. R., Whitaker, J. F., Schulz, T., Chu, E. S., & Albert, R. K. (2007). Preoperative evaluation of the patient with pulmonary disease. CHEST Journal, 132(5), 1637-1645

49 Faul, F., Erdfelder, E., Lang, A.-G., & Buchner, A. (2007). G*Power 3: A flexible statistical power analysis program for the social, behavioral, and biomedical sciences. Behavior Research Methods, 39, 175-191

50 Pocock, SJ. When (not) to stop a clinical trial for benefit. JAMA. 2005;294(17):2228-2230.

15. APPENDICES

APPENDIX A – Definition of Postoperative Pulmonary Complication[44,46]

Respiratory failure	Postoperative PaO2 <60 mmHg on room air, a ratio of PaO2 to inspired oxygen fraction <300, or arterial oxyhemoglobin saturation measured with pulse oximetry <90% and requiring oxygen therapy.
Suspected pulmonary infection	Treatment with antibiotics for a respiratory infection, plus at least one of the following criteria:<br>o New or changed sputum<br>o New or changed lung opacities on a clinically indicated chest radiograph<br>o Temperature >38.3°C<br>o Leukocyte count >12,000/mm
Pleural effusion	Chest radiograph demonstrating blunting of the costophrenic angle, loss of the sharp silhouette of the ipsilateral hemidiaphragm (in upright position), evidence of displacement of adjacent anatomical structures, or (in supine position) a hazy opacity in one hemithorax with preserved vascular shadows.
Atelectasis	Suggested by lung opacification with shift of the mediastinum, hilum, or hemidiaphragm toward the affected area, and compensatory overinflation in the adjacent nonatelectatic lung.
Pneumothorax	Air in the pleural space with no vascular bed surrounding the visceral pleura.
Bronchospasm	Newly detected expiratory wheezing treated with bronchodilators.
Aspiration pneumonitis	Respiratory failure after the inhalation of regurgitated gastric contents.

Table 1 – Definition of Postoperative Pulmonary Complications – Incidence is the composite incidence of any one or more of the above fatal or non-fatal conditions.

APPENDIX B – Data Collection

Preoperative
Sex
Age
ASA
Height
Weight
Preoperative SpO2
Respiratory infection in the last month
Preoperative Hb
Past History of PONV or Motion Sickness
Active Smoker

Intraoperative
Type of Surgery (Specialty)
Name of Surgery
Emergency Surgery (Yes/No)
Surgical Incision (Peripheral, Abdominal, Thoracic)
Duration of Surgery (min.)
Relaxant Used (Roc/Vec)
Type of GA (Volatile/TIVA/Combined)
Neuraxial Anaesthesia and type
Regional Anaesthesia used and type
Any PONV prophylaxis given
    If yes – what was given.

PACU
Airway Events;
    Any desaturation to SpO2<90%
    Need for manual airway support
    Need for oropharyngeal or nasopharyngeal airway
    Need for reintubation in PACU
    Need for anaesthetist to review the patient
    Unplanned ICU admission
PONV score
    1 – no PONV
    2 – PONV responsive to antiemetics
    3 – PONV unresponsive to antiemetics

Postoperative day 1
QoR-15 score, PPC diagnosis (Yes/No and type)

Postoperative day 2 and 3 (if still inpatient);
PPC diagnosis (Yes/No and type)

Hospital Discharge
Hospital Stay (days)
Documented respiratory infection on discharge summary

Postoperative day 30
Patient reported 'chest infection' requiring antibiotics since operation
Patient reported need for NEW or INCREASED bronchodilator therapy since operation
QoR-15 score
Mortality

APPENDIX C – QoR-15 Score[45]

QoR-15 Patient Survey

Date: _ _ / _ _ / _ _ Study #: ____________

Preoperative ☐ Postoperative ☐

PART A

How have you been feeling in the last 24 hours?

(0 to 10, where: 0 = none of the time [poor] and 10 = all of the time [excellent])

Item													
1. Able to breathe easily	None of the time	0	1	2	3	4	5	6	7	8	9	10	All of the time
2. Been able to enjoy food	None of the time	0	1	2	3	4	5	6	7	8	9	10	All of the time
3. Feeling rested	None of the time	0	1	2	3	4	5	6	7	8	9	10	All of the time
4. Have had a good sleep	None of the time	0	1	2	3	4	5	6	7	8	9	10	All of the time
5. Able to look after personal toilet and hygiene unaided	None of the time	0	1	2	3	4	5	6	7	8	9	10	All of the time
6. Able to communicate with family or friends	None of the time	0	1	2	3	4	5	6	7	8	9	10	All of the time
7. Getting support from hospital doctors and nurses	None of the time	0	1	2	3	4	5	6	7	8	9	10	All of the time
8. Able to return to work or usual home activities	None of the time	0	1	2	3	4	5	6	7	8	9	10	All of the time
9. Feeling comfortable and in control	None of the time	0	1	2	3	4	5	6	7	8	9	10	All of the time
10. Having a feeling of general well-being	None of the time	0	1	2	3	4	5	6	7	8	9	10	All of the time

PART B

Have you had any of the following in the last 24 hours?

(10 to 0, where: 10 = none of the time [excellent] and 0 = all of the time [poor])

Item													
11. Moderate pain	None of the time	10	9	8	7	6	5	4	3	2	1	0	All of the time
12. Severe pain	None of the time	10	9	8	7	6	5	4	3	2	1	0	All of the time
13. Nausea or vomiting	None of the time	10	9	8	7	6	5	4	3	2	1	0	All of the time
14. Feeling worried or anxious	None of the time	10	9	8	7	6	5	4	3	2	1	0	All of the time
15. Feeling sad or depressed	None of the time	10	9	8	7	6	5	4	3	2	1	0	All of the time

APPENDIX D – The ARISCAT Score for PPC risk[46]

	β Regression Coefficients	Score
Age (yr)		
≤50	0	0
51–80	0.331	3
>80	1.619	16
Preoperative Spo_2		
≥96%	0	0
91–95%	0.802	8
≤90%	2.375	24
Respiratory infection in the last month		
No	0	0
Yes	1.698	17
Preoperative anemia (Hb ≤10 g/dl)		
No	0	0
Yes	1.105	11
Surgical incision		
Peripheral	0	0
Upper abdominal	1.480	15
Intrathoracic	2.431	24
Duration of surgery (h)		
<2	0	0
2–3	1.593	16
>3	2.268	23
Emergency procedure		
No	0	0
Yes	0.768	8

*Three levels of risk were indicated by the following cutoffs: <26 points, low risk; 26–44 points, moderate risk; and ≥45 points, high risk.

APPENDIX E – The Apfel Score for PONV

Risk factors	Points
Female gender	1
Nonsmoker	1
History of PONV and/or motion sickness	1
Postoperative opioids	1
Sum =	0 … 4

Apfel CC et al. *Anesthesiology*. 1999;91:693.

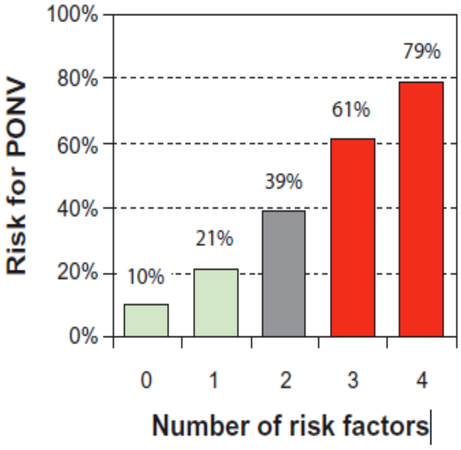

APPENDIX F – PISC Form

Patient Sticker

Patient Trial Number:

APPENDIX G – Study data sheets

P-PERSoN TRIAL – Participant Data Sheet

INSTRUCTIONS TO TREATING ANAESTHETIST

1. On day of surgery, confirm that the patient;
   a. has been spoken to about the study
   b. has been given written information about the study
   c. is happy they have had adequate time to read the information
   d. is happy they understand the written information
   e. is happy to participate in the study

2. Reconfirm the presence of inclusion criteria and the absence of exclusion criteria

3. Confirm signed consent (attached to this data pack)

4. Confirm best telephone number for follow up phone call

   Telephone Number:

5. Obtain Baseline data (page x)

6. Proceed to surgery

The limitations of this study are that you are limited to rocuronium or vecuronium for reversal and that you must use NMT twitch monitoring to ensure return of two twitched prior to reversal. Please review the baseline questions prior to induction (page x)

5. Post Induction
   - Liase with second anaesthetist to receive the patient study number and blinded reversal drug
   - Complete intraoperative data sheet(page x)
   - Annotate inpatient notes with the following;
     - "This patient is involved in a study comparing modes of reversal under anaesthesia (Patient number ###), please contact the duty or on call anaesthetist if you are concerned about the clinical care of this patient being related to invovement in the study"

6. At completion of surgery

At time of administration of reversal (as per you normal practice), confirm recovery of TOFC greater than or equal to 2, administer reversal drug (give when you would normally give the earlier of neostigmine or sugammadex). If TOFC has not returned to greater than or equal to 2, you need to wait for this to occur.

The dose to administer has been standardised to 1mL per 10kg. This will be either 2mg/kg sugammadex or 50mcg/kg neostigmine/10mcg/kg glycopyrrolate.

7. Handover to recovery nursing staff to complete PACU Data sheet. Please enter the Patient Trial Number on the PACU data sheet prior to handing over of care.

Additional Information;

The randomisation envelope will be resealed and kept with the patient notes during theatre and PACU. If you have any clinical concerns about the patient that requires you to break blinding, you can open the envelope. We recommend discussion with the site investigator for the study prior to unblinding.

If you do break blinding, please comment on the reason for this (page x)

If the patient unexpectedly is not extubated at the end of the case and travels to ICU directly, please tick the box on the intraoperative data form and leave the forms in the PACU.

Patient Trial Number:

BASELINE DATA COLLECTION – Treating Anaesthetist prior to induction

Name of Treating Anaesthetist:

Sex	Male Female
Age (years)	
ASA	1 2 3 4 5
Height (cm)	
Weight (kg)	
Preoperative SpO_2 on room air (%)	
Respiratory infection in the last month?	Yes No
Preoperative Haemoglobin (g/L)	
Past History of PONV or motion sickness	Yes No
Current smoker	Yes No

Patient Trial Number:

INTRAOPERATIVE DATA COLLECTION – Treating Anaesthetist during operation

Surgical Specialty	
Name of Surgery performed	
Emergency Surgery	Yes No
Surgical Incision	Peripheral Abdominal Thoracic
Duration of Surgery (minutes)	
Relaxant Used	Rocuronium Vecuronium
Type of GA	Volatile TIVA Combined
Neuraxial Technique Used?	Yes No
PONV Prophylaxis given	Yes No
If Yes – what was given?	

☐ Tick this box to confirm that study drug was administered as per the study protocol. (If not, please document why not – page x)

☐ This patient was admitted to ICU directly and did not travel through the PACU

Definitions;
Emergency Surgery – Unplanned surgery for management of a diagnosis made while in hospital, or for which a diagnosis was made outside of hospital leading to immediate admission to hospital for management. The patient could not be discharged without the surgical procedure

Elective Surgery – Planned surgery to manage a diagnosis that has been known for a period of time. The diagnosis was made outside the hospital and did not result in immediate admission to hospital. The patient could be discharged home without the procedure and rescheduled at a later date.

P-PERSoN Trial

Confirmation of protocol

I can confirm that the treating anaesthetist remained blinded to reversal agent ☐

I can confirm that this patient was treated as per the study protocol ☐

Patient Trial Number:

If not, please describe below any break in protocol, or a break in blinding;

POSTOPERATIVE UNIT DATA COLLECTION – PACU Nursing Staff

Did ANY of the following occur during the patient's stay in the PACU?

Desaturation to SpO2<90%	Yes No
Need for manual airway support	Yes No
Need for oropharyngeal or nasopharyngeal airway	Yes No
Need for reintubation in PACU	Yes No
Need an unplanned/requested review of the patient by an anaesthetist for ANY reason	Yes No
If review needed, briefly state reason	
Unplanned ICU admission	Yes No

Please give the patient's PONV (Postoperative Nausea and Vomiting) score;

1 2 3

1 – no PONV
2 – PONV responsive to antiemetics
3 – PONV unresponsive to antiemetics

Thank you for completing these questions

PLEASE PLACE THE DATA SHEETS IN THE P-PERSON TRIAL AREA IN RECOVERY FOR COLLECTION BY THE TRIAL CLINICAL STAFF

Patient Trial Number:

Postoperative day 1

QoR-15 score -

PPC diagnosis

None ☐

Respiratory Failure ☐

Suspected Pulmonary Infection ☐

Pleural Effusion ☐

Atelectasis ☐

Pneumothorax ☐

Bronchospasm ☐

Aspiration Pneumonitis ☐

Respiratory failure	Postoperative PaO2 <60 mmHg on room air, a ratio of PaO2 to inspired oxygen fraction <300, or arterial oxyhemoglobin saturation measured with pulse oximetry <90% and requiring oxygen therapy.
Suspected pulmonary infection	Treatment with antibiotics for a respiratory infection, plus at least one of the following criteria:<br>o New or changed sputum<br>o New or changed lung opacities on a clinically indicated chest radiog<br>o Temperature >38.3°C<br>o Leukocyte count >12,000/mm
Pleural effusion	Chest radiograph demonstrating blunting of the costophrenic angle, loss of the sharp silhouette of the ipsilateral hemidiaphragm (in upright position), evidence of displacement of adjacent anatomical structures, or (in supine position) a hazy opacity in one hemithorax with preserved vascular shadows.
Atelectasis	Suggested by lung opacification with shift of the mediastinum, hilum, or hemidiaphragm toward the affected area, and compensatory overinflation in the adjacent nonatelectatic lung.
Pneumothorax	Air in the pleural space with no vascular bed surrounding the visceral pleura.
Bronchospasm	Newly detected expiratory wheezing treated with bronchodilators.
Aspiration pneumonitis	Respiratory failure after the inhalation of regurgitated gastric contents.

Patient Trial Number

Postoperative day 2 (if still inpatient);

PPC diagnosis

None ☐

Respiratory Failure ☐

Suspected Pulmonary Infection ☐

Pleural Effusion ☐

Atelectasis ☐

Pneumothorax ☐

Bronchospasm ☐

Aspiration Pneumonitis ☐

Respiratory failure	Postoperative PaO2 <60 mmHg on room air, a ratio of PaO2 to inspired oxygen fraction <300, or arterial oxyhemoglobin saturation measured with pulse oximetry <90% and requiring oxygen therapy.
Suspected pulmonary infection	Treatment with antibiotics for a respiratory infection, plus at least one of the following criteria:<br>o New or changed sputum<br>o New or changed lung opacities on a clinically indicated chest radiog<br>o Temperature >38.3°C<br>o Leukocyte count >12,000/mm
Pleural effusion	Chest radiograph demonstrating blunting of the costophrenic angle, loss of the sharp silhouette of the ipsilateral hemidiaphragm (in upright position), evidence of displacement of adjacent anatomical structures, or (in supine position) a hazy opacity in one hemithorax with preserved vascular shadows.
Atelectasis	Suggested by lung opacification with shift of the mediastinum, hilum, or hemidiaphragm toward the affected area, and compensatory overinflation in the adjacent nonatelectatic lung.
Pneumothorax	Air in the pleural space with no vascular bed surrounding the visceral pleura.
Bronchospasm	Newly detected expiratory wheezing treated with bronchodilators.
Aspiration pneumonitis	Respiratory failure after the inhalation of regurgitated gastric contents.

Patient Trial Number:

Postoperative day 3 (if still inpatient);

PPC diagnosis

None ☐

Respiratory Failure ☐

Suspected Pulmonary Infection ☐

Pleural Effusion ☐

Atelectasis ☐

Pneumothorax ☐

Bronchospasm ☐

Aspiration Pneumonitis ☐

Respiratory failure	Postoperative PaO2 <60 mmHg on room air, a ratio of PaO2 to inspired oxygen fraction <300, or arterial oxyhemoglobin saturation measured with pulse oximetry <90% and requiring oxygen therapy.
Suspected pulmonary infection	Treatment with antibiotics for a respiratory infection, plus at least one of the following criteria:<br>o New or changed sputum<br>o New or changed lung opacities on a clinically indicated chest radiog<br>o Temperature >38.3°C<br>o Leukocyte count >12,000/mm
Pleural effusion	Chest radiograph demonstrating blunting of the costophrenic angle, loss of the sharp silhouette of the ipsilateral hemidiaphragm (in upright position), evidence of displacement of adjacent anatomical structures, or (in supine position) a hazy opacity in one hemithorax with preserved vascular shadows.
Atelectasis	Suggested by lung opacification with shift of the mediastinum, hilum, or hemidiaphragm toward the affected area, and compensatory overinflation in the adjacent nonatelectatic lung.
Pneumothorax	Air in the pleural space with no vascular bed surrounding the visceral pleura.
Bronchospasm	Newly detected expiratory wheezing treated with bronchodilators.
Aspiration pneumonitis	Respiratory failure after the inhalation of regurgitated gastric contents.

Patient Trial Number:

Postoperative day 30 (+/- 5 days)

QoR-15 score –

Patient reported 'chest infection' requiring antibiotics since operation

Yes No

Patient reported need for NEW or INCREASED bronchodilator therapy since operation

Yes No

Hospital Stay (post-surgery days) – recorded from patient notes -

Day of surgery = day 0

Documented respiratory infection and/or aspiration, respiratory failure or exacerbation of chronic respiratory disease on discharge summary -

Yes No

Any of the following on discharge summary –

Myocardial Infarction	Yes	No
Acute Renal Failure	Yes	No
Stroke	Yes	No
Anaphylaxis	Yes	No
Seizure	Yes	No

Mortality

Yes No

APPENDIX H – Study randomisation sheets (in randomised envelope)

Instructions for Unblinded anaesthetist;

This patient has been randomised to receive;

NEOSTIGMINE/GLYCOPYRROLATE

In a 10mL syringe, draw up 5mg of neostigmine and 1mg glycopyrrolate. Use Normal Saline to dilute this to 10mL total volume (500mcg/mL neostigmine and 100mcg/mL glycopyrrolate).

If the patient weighs over 100kg, two syringes should be provided. Please label the syringe with;

"Reversal agent, P-Person trial Participant Number ###"

Instruct the treating anaesthetist to give 1mL per 10kg body weight (rounded to nearest 10kg)

Instructions for Unblinded anaesthetist;

This patient has been randomised to receive;

SUGAMMADEX

In a 10mL syringe, draw up 200mg of sugammadex. Use Normal Saline to dilute this to 10mL total volume (20mg/mL sugammadex).

If the patient weighs over 100kg, two syringes should be provided. Please label the syringe with;

"Reversal agent, P-Person trial Participant Number ###"

Instruct the treating anaesthetist to give 1mL per 10kg body weight (rounded to nearest 10kg)
